# Preparation of blood samples for electron microscopy: The standard protocol

**DOI:** 10.1016/j.amsu.2021.102895

**Published:** 2021-10-05

**Authors:** Om Prakash Choudhary, Rupan Sarkar, Priyanka, G.E. Chethan, Probal Jyoti Doley, Pranab Chandra Kalita, Arup Kalita

**Affiliations:** a Department of Veterinary Anatomy and Histology, College of Veterinary Sciences and Animal Husbandry, Central Agricultural University (I), Selesih, Aizawl, 796015, Mizoram, India; b Independent Researcher, 07, Type IV Quarter, College of Veterinary Sciences and Animal Husbandry, Central Agricultural University (I), Selesih, Aizawl, 796015, Mizoram, India; c Department of Veterinary Medicine, College of Veterinary Sciences and Animal Husbandry, Central Agricultural University (I), Selesih, Aizawl, 796015, Mizoram, India

**Keywords:** Blood, Electron microscope, Red blood cells, White blood cells, Platelets, Karnovasky's fixative, Osmium tetroxide, Phosphate buffer saline

## Abstract

Electron microscopy is a powerful tool to study biological samples at higher magnification. The higher magnifications achieved by the electron microscopes are helpful to the researchers to study surface morphology as well as cellular morphology of the samples. The blood sample surface morphology can be visualized at higher magnification by scanning electron microscope (SEM). For the examination of the blood cells at the cellular level, transmission electron microscopes (TEM) are used. In this article, we have described the step-by-step standard protocol for the preparation of blood samples for electron microscopy. The prepared blood samples can be visualized under SEM and TEM. The obtained electron micrographs of blood cells can be used for differential diagnosis of various diseases at the cellular level.

Electron microscopy of the blood is employed to study the blood cells at higher magnification using the scanning and transmission electron microscopes. This article aims to describe the standard sample preparation protocol for the electron microscopy of the blood cells. Red blood cells (erythrocytes) carry oxygen from the lungs to the rest of the body. White blood cells (leukocytes) help fight infections and aid in the immune process. The white blood cells are categorized as the lymphocytes, monocytes, eosinophils, basophils and neutrophils. Apart from these, the blood cells named platelets (thrombocytes) help in the blood clotting mechanism.

The studies on the blood cells are important from the morphological, physiological, clinicopathological and therapeutic point of view. Examination of blood is important for assessing the general health and diagnosis of various diseases. The blood examination is performed routinely to assess the health status, diagnose haematological diseases, determine the body's ability to respond to a hematological insult and to monitor the course of certain diseases [1]. Various blood cells visualized by the electron microscopy have been demonstrated in Fig. 1. The ultrastructural anatomy of the blood cells is helpful in the diagnosis of several diseases by the examination of blood cells' morphology at higher magnification using electron microscopes.

**Fig. 1:**
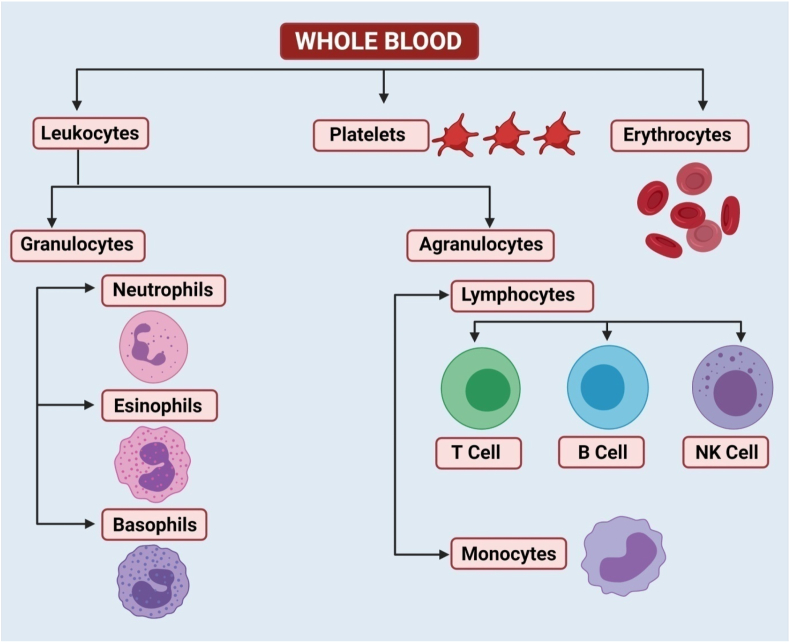
Diagrammatic representation of various blood cells in the humans and animals.

The first step in the sample preparation protocol is the collection of five milliliters (5 ml) of blood in the test tube containing ethylenediaminetetraacetic acid (EDTA) or heparin to prevent blood coagulation. For scanning electron microscopy, the blood is transferred into the Eppendorf tubes and centrifuged at 3000 rpm for 15–20 min. The centrifugation separates the blood into three layers i.e. plasma (55% of total blood), buffy coat (less than 1% of total blood), and red blood cells or erythrocytes (45% of total blood). After discarding the plasma layer from the top of the Eppendorf tube, the thin layer of buffy coat is transferred into a separate Eppendorf tube with the help of a micropipette. This separated buffy coat sample is washed three times in 0.1 M phosphate buffer saline (pH 7.2). The washed sample is suspended in modified Karnovasky's fixative for 2 to 3 h at 4 °C for primary fixation. Subsequently, the suspended buffy coat sample in fixative is washed again in 0.1 M phosphate buffer saline (pH 7.2) to remove the excess amount of primary fixative i.e., modified Karnovasky's fixative. These fixed buffy coats with few attached red blood cells (Fig. 2A) are suspended in 0.1 M phosphate buffer saline (pH 7.2) and transferred to the electron microscopy laboratory for further processing of the blood (buffy coat) for electron microscopy. Researchers can also send the collected blood directly fixed in the modified Karnovasky's fixative at 4 °C to the electron microscopy laboratory for further processing, however this speedy method will not give good results in the electron microscopy. The electron microscopy laboratory's personnel prepare the buffy coat's smear on the slide and visualize it under the scanning electron microscope for higher magnification (approximately up to 1 to 2 million times) of the different blood cells, as shown in Fig. 3 [2,3].

**Fig. 2:**
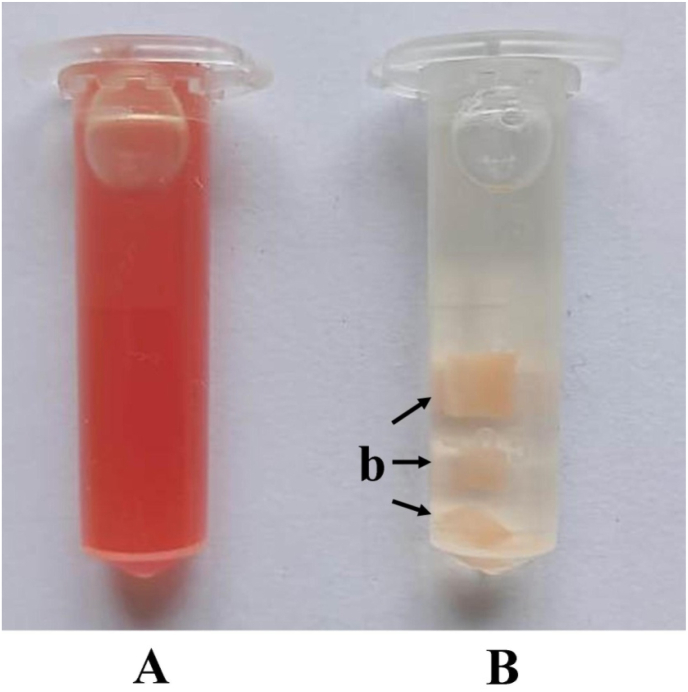
Processed fixed blood sample for scanning electron microscopy (A) and processed blood sample in the pellet form (b) for transmission electron microscopy (B).

**Fig. 3:**
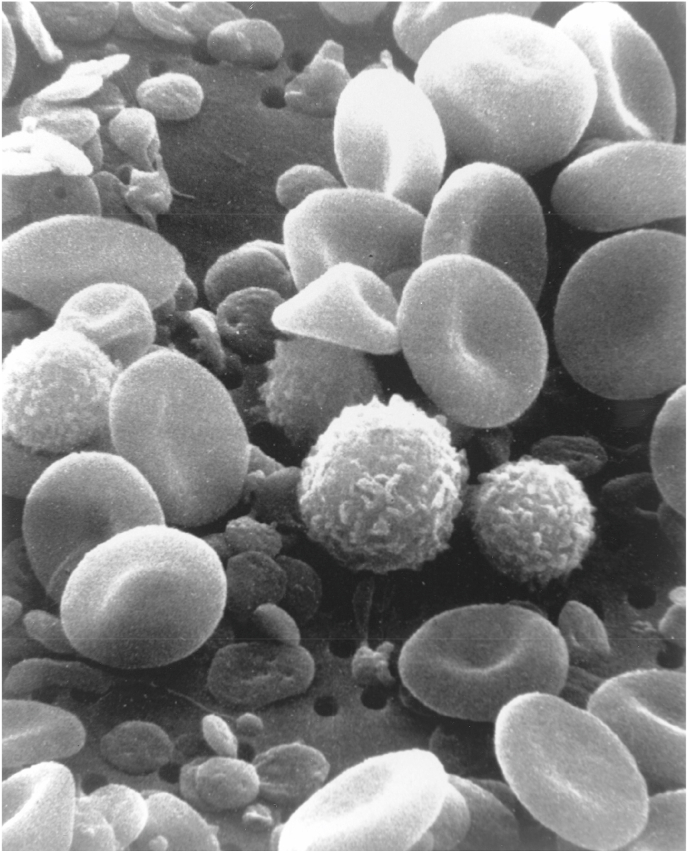
Various blood cells under scanning electron microscope [2].

For transmission electron microscopy, the process of blood collection and centrifugation is the same as the one described for performing scanning electron microscopy. After centrifugation, the excess amount of plasma is removed and the buffy coat is separated in another Eppendorf tube and an equal amount of modified Karnovasky's fixative is poured for primary fixation. The fixed buffy coat is kept at 4 °C in the refrigerator for 12–24 h. After the stipulated period, the buffy coat gets transformed into a semisolid state. The semisolid buffy coat is shifted in the Petri dish for cutting it into small sections (2 mm × 2 mm), as recommended for the transmission electron microscopy [4]. The sections should be washed with the 0.1 M phosphate buffer saline (pH 7.2) solution and then transferred to another clean Eppendorf tube containing 0.1 M phosphate buffer saline (pH 7.2). These processed buffy coat sectioned samples (Fig. 2B) are sent to the electron microscopy laboratory in iceboxes within 24 h. At electron microscopy laboratory, these samples are processed by secondary fixation (2% osmium tetroxide), washing (0.1 M phosphate buffer saline), dehydration (graded alcohol), embedding (resins) and sectioning (ultramicrotome).

Finally, the thin sections are stained with uranyl acetate and examined under a transmission electron microscope [5], which can magnify samples by approximately 50 million times [6]. The images can be taken with an electron microscope digital camera system attached to the electron microscope machine. The images of blood cells captured with transmission electron microscope machine TECNAI 200 Kv (FEI Electron Optics) at All India Institute of Medical Sciences, New Delhi have been shown in Fig. 4.

**Fig. 4:**
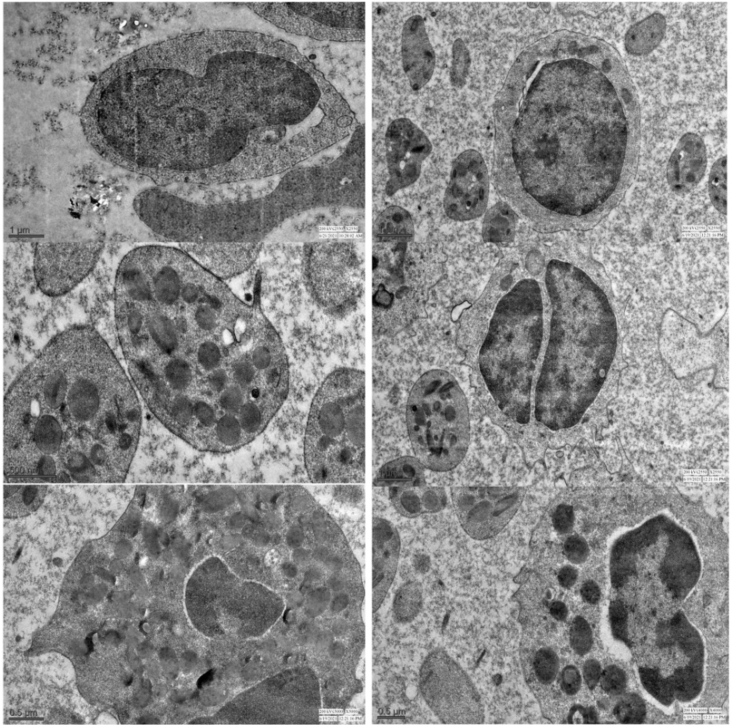
Various blood cells of the local pig of Mizoram under transmission electron microscope.

## Sources of funding

This is part of the methodology of the Intramural Research Project (IRP) entitled “Light and ultrastructural studies on the blood cells of local pig (Zovawk) of Mizoram” funded by Central Agricultural University, Imphal (Code No. Vety.IRP-I/2021–22) under the first author as Principle Investigator (PI).

## Ethical approval

All the procedures involving sample collection were conducted as per the Institutional Animal Ethics Committee (IAEC), which is under the Committee for the Purpose of Control and Supervision of Experiments on Animals (CPCSEA), Ministry of Environment, Forest and Climate Change, Government of India for the College of Veterinary Sciences and Animal Husbandry, Selesih, Aizawl, Mizoram, Aizawl, Mizoram.

## Consent

Not applicable.

## Author contribution

**Om Prakash Choudhary:** Conceptualization, Data Curation, Visualization, Project administration; Funding acquisition, Writing - Original Draft, Writing - review & editing. **Rupan Sarkar:** Methodology, Writing - review & editing. **Priyanka:** Software, Writing - review & editing, **GE Chethan:** Methodology, Writing - review & editing, **Probal Jyoti Doley:** Writing - Original Draft, Writing - review & editing. **Pranab Chandra Kalita**: Writing - review & editing. **Arup Kalita:** Writing - review & editing.

## Registration of research studies

1. Name of the registry: Not applicable
2. Unique Identifying number or registration ID: Not applicable
3. Hyperlink to your specific registration (must be publicly accessible and will be checked): Not applicable

## Guarantor

Om Prakash Choudhary, Assistant Professor, Department of Veterinary Anatomy and Histology, College of Veterinary Sciences and Animal Husbandry, Central Agricultural University (I), Selesih, Aizawl-796015, Mizoram, India. Tel: +91-9928099090; Email: dr.om.choudhary@gmail.com.

## Declaration of competing interest

All authors report no conflicts of interest relevant to this article.
